# Phylogeny and biogeography of *Primula* sect. *Armerina*: implications for plant evolution under climate change and the uplift of the Qinghai-Tibet Plateau

**DOI:** 10.1186/s12862-015-0445-7

**Published:** 2015-08-16

**Authors:** Guangpeng Ren, Elena Conti, Nicolas Salamin

**Affiliations:** Department of Ecology and Evolution, Biophore, University of Lausanne, 1015 Lausanne, Switzerland; Swiss Institute of Bioinformatics, Quartier Sorge, 1015 Lausanne, Switzerland; State Key Laboratory of Grassland Agro-Ecosystem, School of Life Science, Lanzhou University, Lanzhou, 730000, Gansu China; Institute for Systematic Botany, University of Zurich, Zollikerstrasse 107, 8008 ZURICH, Switzerland

## Abstract

**Background:**

The historical orogenesis and associated climatic changes of mountain areas have been suggested to partly account for the occurrence of high levels of biodiversity and endemism. However, their effects on dispersal, differentiation and evolution of many groups of plants are still unknown. In this study, we examined the detailed diversification history of *Primula* sect. *Armerina*, and used biogeographic analysis and macro-evolutionary modeling to investigate a series of different questions concerning the evolution of the geographical and ecological distribution of the species in this section.

**Results:**

We sequenced five chloroplast and one nuclear genes for species of *Primula* sect. *Armerina*. Neither chloroplast nor nuclear trees support the monophyly of the section. The major incongruences between the two trees occur among closely related species and may be explained by hybridization. Our dating analyses based on the chloroplast dataset suggest that this section began to diverge from its relatives around 3.55 million years ago, largely coinciding with the last major uplift of the Qinghai-Tibet Plateau (QTP). Biogeographic analysis supports the origin of the section in the Himalayan Mountains and dispersal from the Himalayas to Northeastern QTP, Western QTP and Hengduan Mountains. Furthermore, evolutionary models of ecological niches show that the two *P. fasciculata* clades have significantly different climatic niche optima and rates of niche evolution, indicating niche evolution under climatic changes and further providing evidence for explaining their biogeographic patterns.

**Conclusion:**

Our results support the hypothesis that geologic and climatic events play important roles in driving biological diversification of organisms in the QTP area. The Pliocene uplift of the QTP and following climatic changes most likely promoted both the inter- and intraspecific divergence of *Primula* sect. *Armerina.* This study also illustrates how niche evolution under climatic changes influences biogeographic patterns.

**Electronic supplementary material:**

The online version of this article (doi:10.1186/s12862-015-0445-7) contains supplementary material, which is available to authorized users.

## Background

Understanding the processes that shape geographical and ecological distribution of biodiversity is one of the most challenging questions in evolutionary biology and ecology. This is particularly true for regions that have experienced rapid habitat changes and harbor high species diversity. These characteristics are present in many mountainous areas and historical orogenesis has been proposed to play an important role in shaping their current biodiversity [1–3]. The alteration of topography and climatic changes associated with mountain uplifts can cause fragmentation of species distributions, thus limiting gene flow between isolated populations and initiating allopatric divergence and speciation [4–7]. However, extreme environmental changes and fragmented distributions can also lead to the extinction of lineages and species (*e.g.,* [8, 9]). The processes occurring during mountain uplifts are therefore complex and we need to better understand the mechanisms that are at play during these events.

The fragmentation of species distributions can be due to the presence of limits on dispersal due, for example, to geographical barriers. Such limitations can induce a reduction in the movement of individuals into new locations and will result in distinct biogeographic patterns in the extant species [10]. However, fragmentation can also occur because of a lower success of establishment of individuals in some areas, which will limit the range of species [11]. This process is primarily set by ecological factors, potentially including both abiotic and biotic variables [10–12]. The dynamics of species range evolution will be constrained by phylogenetic niche conservatism, which is defined as the tendency of species to retain their ancestral ecological niche, thus shaping the geographic ranges of species over time (*e.g.,* [13, 14]). However, evidence for rapid shifts in climatic preferences among species also exists [15, 16] and macro-evolutionary modeling should be used to characterize the processes driving the evolution of ecological niches [17]. A complete assessment of these processes, coupled with detailed analyses of biogeographic patterns of species distribution, should then be used to help understand the distribution of species diversity [10].

One region that experienced drastic habitat changes and harbors extremely rich species diversity and endemism is the Qinghai-Tibet Plateau (QTP; [18]). While the start of its uplift dates from approximately 50 million years ago (Ma; [19]), the extensive uplifts of the QTP occurred in at least four periods since the early Miocene, specifically between 25–17 Ma, 15–13 Ma, 8–7 Ma, and 3.4-1.6 Ma [9, 20–23]. At present, the QTP, with an average altitude of more than 4000 m (a.s.l.), is the highest and one of the most extensive plateaus on Earth [20]. About 9,000–12,000 species of vascular plants in ca. 1,500 genera are present in this plateau, and at least 20 % of these species and ca. 50 genera are endemic [3, 18]. The historical sequence of uplifts of the QTP has been suggested to partly account for the occurrence of high levels of biodiversity and endemism in the region [24]. However, the potential effects of climatic changes during the Quaternary on the diversification and distribution of many groups of plant species in the QTP are not very well known (see Review [2, 3, 25]).

*Primula* L. (Primulaceae) is one of the genera that exhibit high levels of species diversity in the QTP. The group, with a predominantly northern hemisphere distribution, contains ca. 500 species. About 60 % of the species are present in the QTP and its adjacent regions [26, 27]. Although this genus represents an important floristic element of alpine meadows in the region, it remains unclear whether the uplift of the QTP and the following climatic changes affected its diversification and distribution. In this context, a better understanding of the historical biogeography of key floristic elements of the region is an important way to illuminate the evolutionary history of these organisms in space and time*.* Available studies mainly utilize genus- or family-level phylogenies to elucidate the biogeographic connections between the QTP and neighboring regions [28–32]. However, the presence of a single sample per species hardly provides insights into the biogeographic patterns of species distributions within the QTP. Therefore, sampling multiple individuals per species and focusing on endemic species may help to better understand the mechanisms that were responsible for biogeographic patterns within the QTP.

**Fig. 1:**
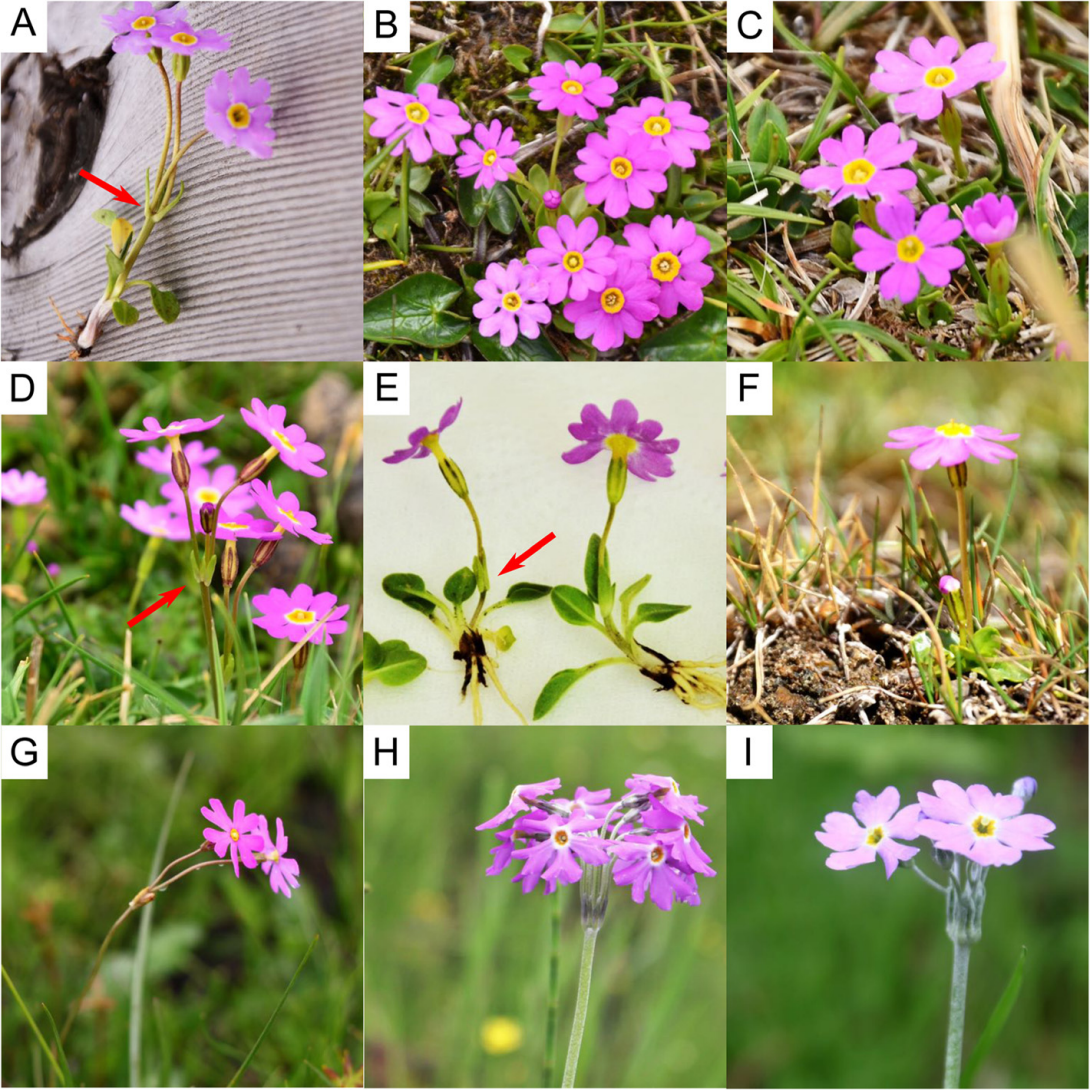
The five species of sect. *Armerina* which showed mainly incongruence between the two trees. (**a**) *P. fasciculata* with linear and non-pouched bracts, (**b**) *P. fasciculata* without bracts, (**c**) one photo of *P. fasciculata* collected from populations of clade F2 (see Results), (**d**) *P. tibetica* with oblong and pouched bracts at low altitude, (**e**) and (**f**) *P. tibetica* with and without bracts at high altitude, respectively, (**g**) *P. nutans*, (**h**) *P. gemmifera*, (**i**) *P. conspersa.* Bracts for *P. fasciculata* and *P. tibetica* are indicated by red arrows. All photos were taken by the first author in the field

In this study, we include several samples per species to investigate the historical biogeography of *Primula* sect. *Armerina* Lindley (Primulaceae), which exhibits a typical Sino-Himalayas distribution. According to the most recent global monographic treatment of the genus, *Primula* sect. *Armerina* comprises 14 species [26]. Eight species (*P. fasciculata, P. tibetica, P. conspersa, P. gemmifera, P. zambalensis, P. pumilio. P. pamirica* and *P. involucrata*; Fig. 1) are endemic to the QTP, with different geographic distributions [26, 27]. Among them, there has been some confusion between *P. tibetica* and *P. fasciculata* because of their morphological similarities at high altitude ([26, 27]; field observation). The two species can be easily distinguished when bracts are present. *Primula tibetica* has oblong and pouched bracts, while the bracts of *P. fasciculata* are linear and non-pouched (Fig. 1a, d). However, at high altitude, bracts are usually missing in *P. fasciculata* (Fig. 1b, c), while in *P. tibetica*, they can also be absent in small individuals with single flower (Fig. 1e, f). Both species have wide altitude distributions, ranging from 2900 m to 5000 m [26, 27] and the use of molecular data combined with macro-evolutionary modeling may provide useful insights into the dynamics of their range evolution. The four remaining species of this section (*P. iljinskyii, P. chrysostoma, P. knorringiana* and *P. valentinae*) have very restricted areas in regions adjacent to the QTP. *Primula nutans* has the most widespread distribution in the section, including N Europe, W & E Siberia, NW America to N Mongolia, NW China and NW QTP. All species from sect. *Armerina* are considered to be diploid (2*n* = 18, 20 or 22) [26, 27], except *P. egaliksensis*, which is the only tetraploid species (2*n* = 36, 40) and occurs mainly in North America. It was assigned to sect. *Armerina* based on morphological features [33, 34], and might be of hybrid origin between *P. mistassinica* (sect. *Aleuritia*) and *P. nutans* [35–37].

Most species of the *Armerina* section are thus prominent floristic elements of alpine meadows at high altitudes in the QTP and most are endemic to the QTP and its adjacent regions [26, 27]. This section of *Primula* hence represents a good candidate to assess the biogeographic history of the QTP and to understand the effects of its uplift and associated climatic changes on the geographical distribution of biodiversity. We analyzed both nuclear and chloroplast DNA sequences of multiple samples per species in the *Armerina* section to reconstruct a comprehensive phylogenetic tree of this group. The aims of our study are to: i) test the inter-specific relationships of sect. *Armerina* to obtain a detailed and resolved phylogenetic tree for the section; ii) assess whether the diversification of this section was influenced by the uplifts of the QTP; iii) combine biogeographic analyses with macro-evolutionary modeling of ecological niches to better understand the role of dispersal and ecological constraints during the diversification of the three main species in the section (*P. fasciculata, P. nutans* and *P. tibetica*).

## Results

### Sequence characteristics

Five chloroplast (*mat*K, *rpl*16, *rps*16, *trn*LF and *trn*H-*psb*A) and one nuclear (translin family protein, *tfp*) markers were sequenced in this study for phylogenetic analyses. The *matK* dataset comprised 892 characters, 815 of which were constant, 22 variable but parsimony-uninformative, 55 variable and parsimony-informative. The *rpl*16 dataset comprised 1063 characters, 903 of which were constant, 90 variable but parsimony-uninformative, 70 variable and parsimony-informative. The *rps*16 dataset comprised 877 characters, 789 of which were constant, 24 variable but parsimony-uninformative, 64 variable and parsimony-informative. The *trn*LF dataset comprised 968 characters, 840 of which were constant, 54 variable but parsimony-uninformative, 74 variable and parsimony-informative. The *trn*H-*psb*A dataset comprised 629 characters, 512 of which were constant, 46 variable but parsimony-uninformative, 71 variable and parsimony-informative. We combined the five plastid regions for all subsequent analyses, modeling them as five partitions. It was not possible to obtain these sequences for *P. watsonii* and four chloroplast sequences (*mat*K-DQ378314, *rpl*16-DQ378443, *rps*16-FJ786584 and *trn*LF-FJ794215) were downloaded from GenBank for this species.

The aligned nuclear dataset comprised 648 characters, 445 of which were constant, 91 variable but parsimony-uninformative, and 112 variable and parsimony-informative. Despite repeated attempts, the *tfp* sequences for three samples of *P. tibetica*, as well as the sample of *P. pamirica*, *P. pumilio* and two outgroup species (*P. watsonii* and *P. pinnatifida*) failed to amplify. Two copies were identified in the samples of *P. fasciculata*, *P. conspersa* and *P. egaliksensis* and these clones were added to the sequences obtained directly from PCR in subsequent phylogenetic analyses.

### Phylogenetic analyses and molecular dating

**Fig. 2:**
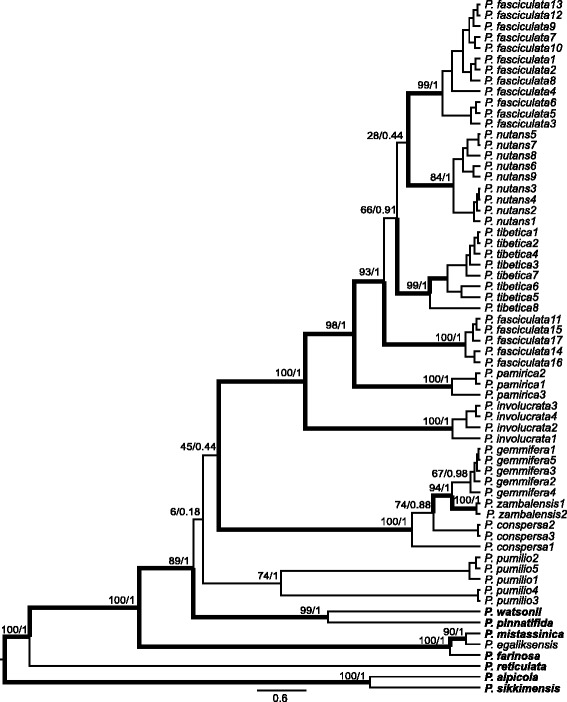
The maximum clade credibility (MCC) tree derived from BEAST analyses of five chloroplast genes. Maximum likelihood (ML) bootstrap values and Bayesian posterior probabilities (PP) are indicated at major nodes. Bootstrap values ≥ 80 and *PP* ≥ 0.95 are indicated with thicker branches. Outgroup species are shown in bold

The maximum likelihood (ML) and Bayesian analyses done on each data set resulted in congruent topologies, but discrepancies were obtained between the two types of markers. The only tetraploid species, *P. egaliksensis,* was included in a well-supported clade with *P. mistassinica* and *P. farinosa* in the chloroplast tree. This result is in agreement with previous studies [35, 37, 38]. The node subtending the rest of the samples of *Primula* sect. *Armerina* received very low support (posterior probability, PP 0.18, ML 6 %) in the choloroplast phylogenetic tree and the relationships between species remained partly unresolved (Fig. 2). Three main clades were inferred in the chloroplast tree. The clade *involucrata* (including *P. involucrata, P. pamirica, P. fasciculata, P. nutans* and *P. tibetica*) and the clade *conspersa* (including *P. conspersa, P. gemmifera and P. zambalensis*) were strongly supported in both ML and Bayesian analyses, while the clade *pumilio* (*P. pumilio*) was not well-supported by ML (74 %), but received very high posterior probabilities in the Bayesian analyses (PP 1.0). Overall, well-supported clades (*PP* > 0.95) in the chloroplast tree grouped sequences from the same species, except for *P. fasciculata*, which was separated into two groups (Fig. 2).

**Fig. 3:**
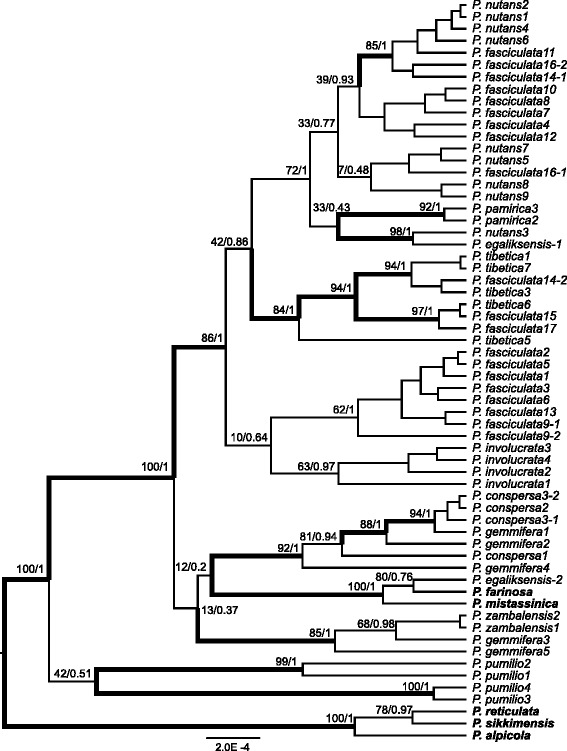
The maximum clade credibility (MCC) tree derived from MrBayes analyses of the nuclear dataset. Maximum likelihood (ML) bootstrap values and Bayesian posterior probabilities (PP) are indicated at major nodes. Bootstrap values ≥ 80 and *PP* ≥ 0.95 are indicated with thicker branches. Outgroup species are shown in bold. Two nuclear gene copies for some samples are indicated with “-1” or “-2”

In contrast to the plastid dataset, *Primula* sect. *Armerina* and two nested outgroup species received very high node support (PP 1.0, ML 100 %) in the nrDNA phylogenetic tree, but the relationships between species were less well supported (Fig. 3). Three main clades within the section identified in the chloroplast tree were also inferred in the nuclear tree (Fig. 3). The clade *involucrata* was well-supported (PP 1.0, ML 86 %), while the clades *conspersa* (except for *P. farinosa, P. mistassinica* and *P. egaliksensis*) and *pumilio* received very weak nodal support in both types of analyses. The relationships within each clade were further incongruent between the trees obtained by the two datasets. *Primula fasciculata* was divided into three clades in the nrDNA tree (Fig. 3). One clade included samples from *P. fasciculata* that cluster with a moderately supported clade representing *P. involucrata*. A second clade included all samples of *P. tibetica* and *P. fasciculata* and one copy of *P. fasciculata*. Finally, the third clade included all samples of *P. nutans* and *P. pamirica*, one copy of *P. egaliksensis* and the remaining samples of *P. fasciculata* (Fig. 3). Similarly, *P. gemmifera* separated into two groups, either with *P. zambalensis* or in a clade including all samples of *P. conspersa* (Fig. 3). Two copies of *P. egaliksensis* were clustered with either *P. nutans* or *P. mistassinica*, corroborating the hypothesis of the allopolyploid origin of this species [35–37].

**Fig. 4:**
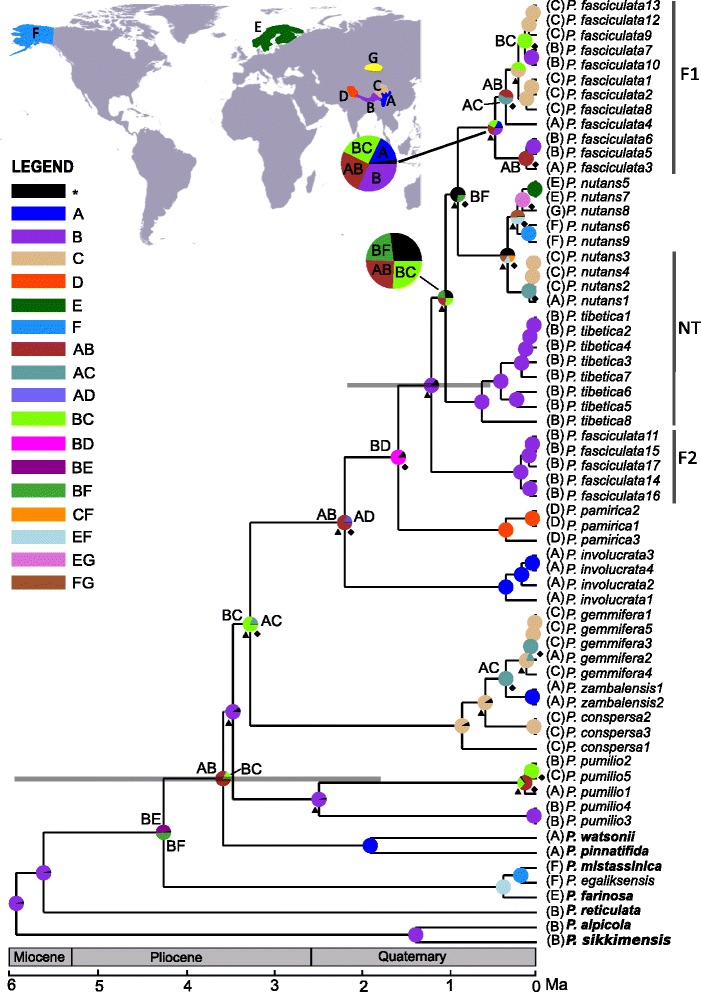
Dispersal–vicariance scenarios for sect. *Armerina* and the outgroup speices based on the chloroplast dataset reconstructed by Statistical Dispersal–Vicariance Analysis (S-DIVA) optimization with the maximum number of area units set to two. Triangle: dispersal event; diamond: vicariance event. Letters denoting area units are indicated on the map. Pie charts at internal nodes represent the marginal probabilities for each alternative ancestral area. Alternative ancestral areas (letters on nodes) are indicated for the major nodes. The grey bars on the nodes represent the 95 % highest posterior density intervals of the dates obtained from BEAST analyses. Time scale is shown at the bottom. Three groups (F1, F2 and NT) are used for the evolutionary niche models: groups F1 and F2 are two clades of *P. fasciculata* in the chloroplast tree; group NT includes all samples of *P. tibetica* and samples of *P. nutans* that were only collected from the QTP

Previous dating analyses at the level of the family used low intra-sectional sampling and suggested that sect. *Armerina* diverged from its relatives about 5 Ma [38]. This date is generally congruent with the results of our dating analysis, which indicated that the section (except *P. egaliksensis*) diverged from its two relatives, *P. watsonii* and *P. pinnatifida,* 3.55 Ma (1.76–5.93 Ma, 95 % highest probability density, HPD; Fig. 3). Most cladogenetic events in this section occurred during the past 3.4 million years (Fig. 4). The crown age of the three closely related species, *P. nutans*, *P. fasciculata* and *P. tibetica*, was about 1.19 Ma (95 % HPD: 0.51–2.13 Ma; Fig. 4).

### Biogeographic inference

Biogeographic analysis based on the chloroplast dataset was reconstructed by Statistical Dispersal–Vicariance Analysis (S-DIVA). Fourteen dispersal and 15 vicariance events for the section were identified in this analysis (Fig. 4). The origin of this section was inferred with high confidence in the Himalayan Mountains (B, 91 %). We found that one clade (*P. zambalensis*, *P. gemmifera* and *P. conspersa*) colonized the Northeast QTP (C) and subsequently diversified and dispersed to the Hengduan Mountains (A), while *P. pamirica* colonized the Mountains of Central Asia (D). The common ancestral area of *P. fasciculata*, *P. tibetica* and *P. nutans* was inferred to be in the Himalayan Mountains (B, 86 %).

### Evolution of ecological preferences

We fitted a series of macro-evolutionary models based on 19 bioclimatic variables (*i.e.,* climatic niches) to better understand the biogeographic patterns of three closely related species, *P. fasciculata*, *P. tibetica* and *P. nutans*. We extracted the 19 bioclimatic variables from the sampled localities of the three species (Additional file 1). For *P. nutans*, we used only the samples that were collected in the QTP. The first two axes of the principal component (PC) analysis based on this dataset explained 53.2 % and 25.3 % of variance, respectively. The first axis (PC1) was strongly and positively correlated with temperature seasonality (BIO4, WorldClim variables) and negatively correlated with temperature in coldest and driest Quarter (BIO6 and BIO9). The second axis (PC2) was correlated strongly and positively with precipitation in coldest and driest Quarter (BIO14, BIO17 and BIO19), and strongly and negatively with precipitation seasonality and mean diurnal range (BIO2 and BIO15).

We used the values obtained for PC1 and PC2 (Additional file 2) to test for the evolution of the ecological niche in *P. fasciculata*, *P. tibetica* and *P. nutans*. The Brownian motion model was rejected for both PC1 and PC2 in all species sets tested (Additional file 3). For PC1, the best-performing models were OU1 for SET1, SET2 and SET3, and OUMV for SET4. Average AICc weights were 0.46, 0.36, 0.66 and 0.48, respectively (see Additional file 3 for all AICc weights). The OUM was the second-best model for SET1 (Average AICc weights = 0.25). The OUMV, OUMA and OUM models that allow different niche optima for SET2 also received non-negligible AICc weights (0.29, 0.18, 0.12). For PC2, all four sets were best modeled under OUMV (AICc weights 0.97, 0.93, 0.78 and 0.64 respectively; Additional file 3).

**Fig. 5:**
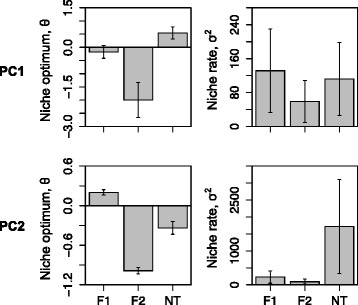
Parameter estimates of models of niche evolution for the three groups (F1, F2 and NT). For PC1, averaged parameters are obtained based on three supported models (OUM, OUMV and OUMA). The averaged strength of selection (α) estimated across models for the three groups is similar and not shown. For PC2, parameter estimates are from the only supported OUMV model (different rates σ^2^ and niche optima θ among the three groups)

The parameters (niche optimum θ, rate of niche evolution σ^2^ and strength of selection α) estimated for the three species groups (F1, F2 and NT) from all supported models based on the four group sets were congruent (Additional file 4) and we showed the parameters estimated based on SET2 (Fig. 5). We used model averaging to estimate the parameter values for PC1 over the supported models OUMV, OUMA and OUM. The averaged niche optima (θ) across models for group F1, F2 and NT were −0.17, −2.0 and 0.55, respectively (Fig. 5). The averaged rate parameter (σ^2^) across models for group F2 was two times slower than that for the groups F1 and NT (59 vs. 131 and 112). Finally, the averaged strength of selection estimated across models for the three groups was similar (6.9, 6.3, 6.9). For PC2, model OUMV, which allows for different niche optima and rates of niche evolution among groups, was the only supported model. The optimum values estimated based on this model for the three groups were also different from each other (F1: 0.2, F2: −0.99, NT: −0.33). The group F2 still exhibited the slowest rate of niche evolution (F1: 228, F2: 94, NT: 1723; Fig. 5).

## Discussion

### Non-monophyly of *Primula* sect. *Armerina*

The phylogenetic analyses of *Primula* sect. *Armerina* presented here contain samples of several individuals per species and cover most of the geographic distributions of the species. Neither the chloroplast tree nor the nuclear tree supports the monophyly of sect. *Armerina*. The section and the two outgroup species, *P. watsonii* and *P. pinnatifida* (sect. *Muscarioides*) form a well-supported clade in the chloroplast tree, despite the fact that these two outgroup species are distinguished from sect. *Armerina* by clear morphological traits (*e.g.,* spicate inflorescence *vs.* umbel; [26, 27]). Similarly, the two outgroup species, *P. farinosa* and *P. mistassinica* (sect. *Aleuritia*), are grouped with the section and form a well-supported clade in the nrDNA tree. The non-monophyly of sect. *Armerina* is in agreement with previous family-wide analyses [38, 39]. Moreover, non-monophyly of sections in genus *Primula* seems pervasive in phylogenetic trees [38, 39].

### Phylogenetic relationships within the section

The relationships among some of the basal nodes of the section in the nuclear tree are uncertain (Fig. 3), which may result from low sequence divergence within the section. The use of a single nuclear gene is thus clearly not sufficient to resolve the relationships within the group, which is a pattern often found also in other lineages (*e.g.,* [40, 41]). Multiple nuclear genes or genomic data are therefore needed to resolve the precise relationships between the main clades in this group. However, both phylogenetic trees show three main clades within sect. *Armerina*, which is in agreement with previous phylogenetic studies [39] as well as morphological based taxonomy [26, 27].

Phylogenetic relationships inferred from the nuclear and chloroplast datasets were incongruent (Figs. 2, 3). The tree obtained from the latter is in agreement with morphology-based taxonomy, which contrasts with other studies that showed a better congruence of taxonomy with the trees inferred from nuclear datasets (*e.g.,* [42]). Incongruence between different plant genomic markers is found in numerous studies and can be explained by incomplete lineage sorting, hybridization and introgression [40, 42–45]. Introgression represents the transfer of genes between species mediated primarily by backcrossing [46], but it does not seem a likely explanation for the incongruence that we observed. Maternally inherited chloroplast loci with relatively low rates of intraspecific gene flow should be more frequently introgressed [46]. In contrast, biparentally inherited nuclear loci that experience high rates of intraspecific gene flow should enhance species delimitation [46]. We find the opposite pattern in our results (Figs. 2 and 3). The chloroplast tree has much clearer species delimitation than the nuclear tree and this pattern seems incompatible with the assumption that the incongruence results from introgression.

Although introgression cannot occur without hybridization, hybridization followed by no backcrossing and introgression could still occur and such phenomenon has been detected in numerous studies (*e.g.,* [47, 48]). Natural hybridization in *Primula* is common and has been confirmed by several studies [37, 49–51], although, it is currently unclear to what degree species within sect. *Armerina* hybridize with each other. The incongruent placement of *P. egaliksensis* between chloroplast and nuclear gene trees can be explained by hybridization [35–37]. Moreover, our results provide further evidence in support of the hypothesis that *P. egaliksensis* originated from an intersectional allopolyploidization event, which places the two *tfp* copies of *P. egaliksensis* with either *P. nutans* or *P. mistassinica* (Fig. 3)*,* confirming previous results by Guggisberg et al. [35–37]. From this perspective, similar incongruence detected for sample number 14 of *P. fasciculata* (two *tfp* copies grouped with either *P. nutans* or *P. tibetica*) may also result from hybridization. Beside hybridization, incomplete lineage sorting is another important explanation for the incongruence between data sets, but the two processes are often difficult to distinguish from each other [52–54]. Although incomplete lineage sorting could also be involved in the incongruences found in our results, the occurrence of such a process would imply that the origin of the haplotypes of *P. pamirica* preceded the speciation events of the whole clade [52]. Such extensive levels of incomplete lineage sorting may yield gene trees with random patterns of relationships among taxa [55]. The patterns of relationships are, however, non-random in the nuclear tree. The major incongruences result mainly from the division of *P. fasciculata* and *P. gemmifera* in different lineages. We thus consider hybridization as the most likely explanation for the major incongruences between chloroplast and nuclear trees.

However, it should be noted that using a single nuclear gene that provides low resolution of the phylogenetic relationships might not be sufficient to elucidate the reasons of the genealogical incongruences between different genomic markers. Although our results tend to suggest a more probable role of hybridization as the most likely explanation for the major incongruence between two trees, incomplete lineage sorting and introgression cannot be completely excluded. We therefore recognize that gene trees/species trees analyses involving multiple nuclear loci or population genomic approaches would be necessary to clearly discriminate among these possible scenarios.

### Biogeographic history

The biogeographic reconstruction based on the chloroplast dataset showed that sect. *Armerina* originated in the Himalayas and subsequently dispersed to the Hengduan Mountains, Northeastern QTP and Western QTP (Fig. 4). The lineages involved in these dispersal events further diversified in the Hengduan Mountains, Northeastern QTP and Western QTP, respectively, and gave rise to several of the extant species. Our dating analysis estimates that this section diverged from its closest relatives in the Pliocene about 3.55 Ma (95 % HPD: 1.76–5.93 Ma, Fig. 4). The timeframe of this event coincides with the recent uplift of the QTP, which occurred between 1.6 and 3.4 Ma [21, 22, 56]. A similar time of divergence was also observed in other groups of plants distributed in the QTP [32, 57–59]. It has been suggested that the uplifts of the QTP might have limited the spread of many species, but accelerated speciation via vicariance [60]. The timeframe of the uplift also coincides with a period of high climatic oscillations that could have reinforced the processes initiated by the uplifts [57, 58].

Vicariance and dispersal triggered by the uplift of the QTP and associated climatic changes are common mechanisms in the diversification of plants in the QTP (*e.g.,* [59, 61]), and also in other mountain areas (*e.g.,* [1, 62, 63]). Based on the S-DIVA analysis, five of the 15 vicariance and seven of the 14 dispersal events account for cladogenetic events, and both events occurred during and after the Pliocene uplift of the QTP (Fig. 4). Vicariance and dispersal triggered by the uplift of the QTP and Quaternary climatic oscillations may accelerate the early diversification of sect. *Armerina*, and further shape the biogeographic patterns [59]. Furthermore, ten “vicariance” (for ease of notation, here we still keep the word “vicariance” for the isolation of populations of the same species) and seven dispersal events are identified within species-specific clades, which might play a role in promoting intraspecific divergence. Extensive inter- and intra-specific divergence took place in the QTP within the Pliocene and Quaternary climatic changes in many groups of plants (*e.g.,* [64–68]). Our analyses together with previous studies thus highlight the importance of the Pliocene uplift of the QTP and Quaternary climatic changes in promoting the diversification of plants in this mountain area.

### Niche evolution of *P. fasciculata*

The S-DIVA analysis shows different biogeographic patterns for the two *P. fasciculata* clades*.* One clade (F2; Fig. 4) occupies only Northern Tibet, while samples from the other clade (F1) can be found in the Hengduan Mountains, Eastern Tibet and Northeastern QTP. Wiens & Donoghue [69] argued that phylogenetic niche conservatism and niche evolution might be critical in the biogeographic history of many groups. In contrast to most previous studies that have suggested the importance of niche conservatism in setting range limits and creating biogeographic patterns (*e.g.,* [70, 71]), niche evolution under climatic changes seems to be the major factor explaining the biogeographic patterns detected here. Although the OU1 model that allows a single niche optimum is the best model along the temperature gradient (PC1), models that allow different niche optima received together higher AICc weights (AICc_OUM+OUMV+OUMA_ = 0.59). This result suggests that ecological differentiation (*i.e.,* different niche optima) is occurring in this group.

The two *P. fasciculata* clades and their two closely related species are estimated to diverge from each other during the Quaternary after the uplift of the QTP (Fig. 4). Climatic oscillations during the Quaternary had a dramatic effect on species distribution ranges [72]. Many species have repeatedly retreated and expanded their distributions following these climatic oscillations (*e.g.,* [57, 58, 72, 73]. In the context of a changing environment, dispersal plays a crucial role in tracking favorable environmental conditions through space [74]. It can also help adaptation of small populations through both demographic and genetic rescue effects [75, 76]. Two dispersal events may have provided the opportunities for populations of clade F1 to occupy wide ranges and also invade new habitats and climatic regimes (Fig. 4). These events are associated with relatively relaxed niches (*i.e.,* niche optima are not strongly correlated with temperature and precipitation gradient; Fig. 5) and fast niche evolution (Fig. 5) and these characteristics might have allowed these populations to adapt to the changing environmental conditions [11, 77]. In contrast, populations of clade F2 occur at higher altitudes (average 4600 m) compared to those of clade F1 (average 4200 m). These populations of clade F2 might have been adapted to a colder climate characterized by lower temperature seasonality (*i.e.,* cooler summers) and less precipitation in the coldest Quarter (see Results of PCA and Fig. 5). Clade F2 displays a lower rate of niche evolution than F1 populations and this lower rate could have limited its dispersal into lower and warmer places. A similar pattern was observed in tropical treefrogs [78], which were unable to extend their ranges further North into temperate regions. Furthermore, recent climatic changes are involved in a shift toward higher elevations in the climatic envelopes of two closely related monkey-flower species in the direction of higher elevations [79]. However, given the harsh environmental conditions, it is plausible that climatic warming in the future might adversely affect the populations of the clade F2 and cause their distributions to shrink [57]. Our results also indicate that contrasting evolutionary processes can occur within closely related lineages, reinforcing the idea that phylogenetic niche conservatism is unlikely to hold at lower spatial scales [80].

While we focus on climatic variables (*i.e.,* temperature and precipitation) to explain the biogeographic patterns detected here, additional ecological factors such as edaphic variables, competition, seed bank and seed number could be involved in creating biogeographic patterns [10, 12]. As argued by Hoskin et al. [81], geographic isolation of populations within species and variation in ecological factors are major precursors to cryptic speciation. The ecological differences and biogeographic patterns found between the two *P. fasciculata* clades may have given rise to some degree of differential adaptation to their respective environmental conditions, as also suggested in *Taxus wallochiana* [58]. However, our data is not appropriate to gain a detailed knowledge of the processes at play here and further studies involving a finer sampling of populations associated with large scale genomic data should be employed to better understand the mechanisms involved in the separation of the *P. fasciculata* clades.

## Conclusion

Our phylogenetic analyses, based on both chloroplast and nuclear datasets, show non-monophyly of *Primula* sect. *Armerina*, corroborating the results of previous family-level studies [38, 39]. The topologies inferred from nuclear gene and concatenated chloroplast datasets are incongruent, which may mainly result from hybridization. This section was suggested to originate in the Himalayas during the Pliocene uplift of the QTP. Subsequent dispersals to the Hengduan Mountains, Northeastern QTP and Western QTP were considered as the consequence of the Pliocene uplift of the QTP and following climatic changes. We further provide a practicable framework for the first time to test the relationship between biogeographic patterns and ecological factors in the QTP area. Our evolutionary models suggest that niche evolution, rather than niche conservatism, seems to explain the biogeographic patterns of the two *P. fasciculata* clades.

## Methods

### Sampling and extraction

We collected in total 57 samples representing 10 of the 14 species belonging to *Primula* sect. *Armerina* (Additional file 5). We could not obtain plant material for *P. iljinskyii, P. chrysostoma, P. knorringiana* and *P. valentinae*, which have small distributions in Central Asian Mountains and are difficult to obtain due to their geographical locations. Widespread species were collected from different localities across their geographical ranges. For example, *P. nutans* was represented by two samples from N America, two from N Europe, one from NW Mongolia and four from China. Seven outgroup species were sampled based on the large phylogenetic tree of Primulaceae (Additional file 5; [38]). All samples were dried and stored in silica gel after collection, except for *P. pamirica*, which was obtained from Harvard University herbaria. The leaf tissues were ground to dust using an electric tissue homogenizer. Total genomic DNA was then isolated using the DNeasy Plant Mini Kit (Qiagen AG, Hombrechtikon, Switzerland) following the manufacturer’s instructions.

### Amplification and sequencing

Five chloroplast DNA regions and one nuclear gene were sequenced. Three cpDNA loci (*rpl*16 intron; *trn*L-F region, which comprises the *trn*L intron and the *trn*L-*trn*F intergenic spacer; *rps*16 intron) were amplified and sequenced using the published primers [34]. The *mat*K gene and *trn*H-*psb*A intergenic spacers were amplified and sequenced following the protocol described in Li et al. [82]. For the nuclear gene, we designed three pairs of exon-primed-intron-crossing (EPIC) primers based on an *Arabidopsis thaliana* translin family protein locus (*tfp*, AT2G03780) and a *Primula sieboldii* seedling cDNA library (FS228429). Only one pair of primers: *tfp*_e1.F (5’-CGAGAAAGGGTGGTAAAAGC-3’) and *tfp*_e1.R (5’-CTGGGGAGTAAGCTCGTCTG-3’), was amplified successfully for sect. *Armerina*. Polymerase chain reactions (PCR) generated double bands and direct sequencing of *tfp*_e1 amplicons produced electropherograms with double peaks and non-complementarity between sequenced strands in the following accessions: *P. fasciculata* (populations 9, 14, 16), *P. conspersa* (population 3) and *P. egaliksensis.* These PCR products were applied on a 1.5 % agarose gel, then excised and purified using a QIAquick Gel Extraction Kit (Qiagen, cat. no. 28704). The purified products were subsequently cloned into a pTZ57R/T vector and sequenced. Eight clones were sequenced per band.

All PCR reactions were performed in 25 μL volumes containing 1 × buffer (including 1.5 mM MgCl_2_), 2 mM MgCl_2_, 300 μM dNTPs, 0.2 μM of each primer and one unit Taq polymerase (GoTag DNA Polymerase, Promega, Madison, WI, USA). Amplifications were carried out on a thermocycler (Biometra, Goettingen, Germany) using the following conditions: a first cycle at 94 °C for 3 min; 36 cycles at 94 °C for 40 s, 55 °C for 1 min and 72 °C for 1.2 min; a final cycle of 7 min at 72 °C. All sequencing reactions used the Big Dye 3.1 Terminator cycle sequencing kit (Applied Biosystems, Foster City, CA, USA), then sequenced on an ABI Prism 3100 genetic analyzer (Applied Biosystems). DNA sequences were aligned with Geneious 6.1.6 (Biomatters) using MAFFT [83] and revised manually. The nuclear gene data generated from direct sequencing were scanned carefully and edited when necessary to ensure that all double peaks were identified correctly with standard degeneracy codes (*e.g.,* Y means C or T; R means G or A; W means A or T; K means G or T; M means C or A). When double peaks were detected at a site, the site was ascertained as ambiguous only if the weakest signal reached at least 25 % of the peak signal strength [84, 85]. For individuals that contained multiple clones for the *tfp* gene, we randomly chose a single representative sequence for the phylogenetic analysis if all the clones formed a well-supported clade in a preliminary analysis, while multiple sequences were retained otherwise. All sequences were submitted to GenBank (accessions KT259477-KT259852).

### Phylogenetic reconstruction and molecular dating

The five chloroplastic genes were concatenated into a single dataset using SequenceMatrix 1.7.8 [86], while the chloroplast and nuclear datasets were analyzed separately. The GTR + G model of sequence evolution was selected on the basis of the Akaike information criterion (AIC) for all DNA regions as estimated by jModelTest 2.1.4 [87]. Maximum likelihood analyses were done with PhyML (ver. 3.0; [88]) using the BEST algorithm for branch swapping and 10^3^ bootstrap replicates to assess node support. We estimated tree topology by Bayesian inference using MrBayes 3.2 [89] with the GTR + G model of evolution and default priors. We unlinked the parameters of the GTR + G model between the five different genes for the analysis of the chloroplast dataset. We repeated the MrBayes analyses three times for each analysis (*i.e.,* chloroplast and nuclear dataset) and each analysis consisted of four chains of 10^7^ generations, sampling every 10^3^ steps with temperature parameter set to 0.1. We determined convergence by examining trace plots of the log-likelihood values for each parameter in Tracer 1.5.

We used the chloroplast dataset for dating analysis with a secondary calibration strategy, as described in de Vos et al. [38], However, age estimation obtained from this kind of calibration may be inherently subjected to bias and errors [90]. We addressed this concern by comparing our estimated age with previously published ones, but it should be noted that they are estimates that should be treated with caution. Divergence time analysis was performed in BEAST (ver. 1.7; [91]). The fossil record of Primulaceae is too sparse to provide multiple and reliable calibrations within the family [92, 93]. The only available fossil that can be used as minimum-age estimate for the split between *Primula* and *Soldanella* is represented by seeds from *Primula riosiae* from the Miocene that are dated at 15.97 Ma (the early-mid Miocene boundary; [94]). Therefore, we performed a completely separate divergence-time analysis from a taxonomically more inclusive sample of six plastid gene regions (*mat*K, *ndh*F, *rbc*L, *trn*L-F, *rps*16 and *rpl*16) available in GenBank (Additional file 6). We included *P. fasciculata*, *P. involucrata*, *P. sikkimensis* and *P. alpicola* in the larger analyses to obtain a root age estimate for *Primula* sect. *Armerina*. The resulting data matrix comprised 7978 aligned sites and 13 species of Primulaceae, with 8.3 % missing data (Additional file 6). Sequence alignment and model specification proceeded as described above, unless otherwise stated. The GTR + G model of sequence evolution was selected by jModelTest 2.1.4 for *rpl*16, *trn*L-F, *ndh*F and *mat*K, GTR + I model for *rps*16 and HKY + G + I model for *rbc*L. A normally distributed prior with a mean of 39.996 Ma and a standard deviation of 11.492 Ma [38] was used to constrain the root of the *Soldanella*/*Androsace* divergence to be within the interval 21.09–58.90 Ma with 95 % probability. The calibration point between *Primula* and *Soldanella* based on the fossil of *Primula riosiae* was set to a lognormal prior with an offset of 15.97, a mean of 2.1 and a standard deviation of 0.63. The analyses were run using a random starting tree for 10^8^ generations sampling every 10^3^ generations under the uncorrelated lognormal relaxed clock model, a birth-death tree prior and the selected models of substitution for different partitions. The analyses were repeated three times to verify convergence by examining the posterior distribution of parameters in Tracer 1.5. After the removal of the burn-in (10 million generations in each analysis, corresponding to 10 % of the samples), the inferred age distribution of the node separating the groups containing either *P. fasciculata* and *P. involucrata* or *P. sikkimensis* and *P. alpicola* was estimated in Tracer 1.5.

The age obtained for the *Armerina* section was then used as a calibration point for the root age of the *Armerina* analysis and modeled as a γ prior with a shape of 9.7, a scale of 0.61 and an offset of 1.4. We used similar settings as described above and the samples retained after removal of the burn-in from the three runs were summarized as a maximum clade credibility tree with mean divergence times using TreeAnnotator (part of the BEAST package).

### Biogeographic reconstruction

We ran Statistical Dispersal Vicariance Analysis (S-DIVA) using RASP v.2.1 [95, 96] to infer the biogeographic history of this section based on the phylogenetic trees constructed only from our concatenated chloroplast dataset. We did not use the *tfp* nuclear dataset since two homologous copies were obtained from some samples, but multiple copies were not present in all species. We defined seven biogeographic regions for the individuals that were collected: A (East Tibet and Hengduan Mountains), B (Himalayas Mountains), C (Northeast QTP), D (Monutains of Central Asia), E (North Europe), F (North America) and G (Mongolian Plateau). Regions A-C were defined according to the biogeographic divisions of China [97], and had been applied in other studies (*e.g.,* [59, 98]). Region D was defined based on the distribution area of *P. pamirica*. Regions E-G were defined based on the distribution of *P. nutans* and some outgroup samples used in this study. To account for uncertainties in phylogenetic reconstructions, we randomly chose 20,000 trees from the posterior distribution of trees obtained by BEAST. The number of maximum areas was set to 2 and we estimated the possible ancestral ranges at each node of the selected phylogenetic trees.

### Evolution of ecological preferences

Climatic niche is one of the main factors for setting historically biogeographic patterns, especially during drastically climatic changes, such as Quaternary climate oscillations [10–12]. In order to better understand the biogeographic patterns obtained above, we fitted a series of macro-evolutionary models based on 19 bioclimatic variables. We focused on the clades formed by the species *P. fasciculata*, *P. tibetica* and *P. nutans* because they represent the main lineages in the group, and tested whether the evolutionary trajectories of the climatic niches differed among the different clades (F1, F2; Fig. 4) obtained for *P. fasciculata* (see Results) and its two closely related species *P. nutans* and *P. tibetica* (NT; Fig. 4). For this test, we used only the samples of *P. nutans* that were collected in the QTP.

We extracted the 19 bioclimatic variables of WorldClim (http://www.worldclim.org/current; [99]) for all samples of the three groups (F1, F2, NT) using the package raster [100] in R. All the 19 bioclimatic variables were then summarized into principle components using the *prcomp* function in the *stats* package of R [101]. We used the R package OUwie [102] to compare the fit of a series of models (see Table 1 for detailed interpretation for each model) to explain the differences in niche evolution between species inhabiting similar or different biogeographic regions. We tested these models on different sets of groups: (1) F1/F2 *vs.* NT (SET1); (2) F1 *vs.* F2 *vs.* NT (SET2); (3) F1 *vs.* F2/NT (SET3); and (4) F2 *vs.* F1/NT (SET4).

**Table 1:** Models of niche evolution relevant to different group-sets with their parameters and interpretation, indicating for each model whether the optimal niche value, θ, the intensity of random fluctuations in the evolutionary trajectory, σ^2^, and the strength of selection toward the optimal value, α, are modeled with one global parameter or with two or three parameters that are group-specific

	Parameters			
Model	θ	σ^2^	α	Interpretation for models
BM1	Global	Global	-	Evolution is random
BMS	Global	Group-specific	-	Different groups have different rates of niche evolution
OU1	Global	Global	Global	Niche evolution is directed toward an optimal value without being affected by different groups
OUM	Group-specific	Global	Global	Different groups have different optimal values
OUMA	Group-specific	Global	Group-specific	Different groups have different optimal values and strength of selection
OUMV	Group-specific	Group-specific	Global	Different groups have different optimal values and rates of niche evolution
OUMVA	Group-specific	Group-specific	Group-specific	Different groups have different optimal values, strength of selection and rates of niche evolution

Stochastic mapping for all model tests were run 10 times for 100 trees randomly selected from the posterior distribution of trees from the BEAST analysis to account for possible uncertainty in the estimated values. Model fit was determined using AICc weights calculated from ΔAICc scores [103]. The highest value of AICc weight represents the best model. Finally, we calculated an average AICc weight and lower (2.5 %) and upper (97.5 %) quantiles of the distributions of AICc weights for each evolutionary niche model.

## Additional files

Additional file 1:
**The 19 bioclimatic variables for each samples of the three groups used for the niche models.** (DOCX 153 kb) Additional file 2:
**The PC1 and PC2 values summarized from the 19 bioclimatic variables used for the niche models.** (DOCX 79 kb) Additional file 3:
**Average AICc weights for each niche evolutionary model.** The values are averages across estimations done on 100 trees with 10 stochastic maps. Quantiles (reported in brackets below each average AICc weight) are calculated as 2.5 % and 97.5 % from the distribution of AICc weights based on 100 trees, with 10 stochastic maps. The bold values show the best-fit models for each set and each PC axis. (DOCX 86 kb) Additional file 4:
**Model fit and estimates parameters of supported OUwie models for the four group-sets.** Parameter estimates are averages across estimations done on 100 trees with 10 stochastic maps. Quantiles (reported in brackets) are calculated as 2.5 % and 97.5 % from the distribution of AICc weights based on 100 trees, with 10 stochastic maps. F1, F2 and NT are three groups defined based on the chloroplast tree (see Fig. 4). (DOCX 119 kb) Additional file 5:
**List of samples used in the present study.** (DOCX 125 kb) Additional file 6:
**GenBank accession numbers for DNA sequence data of the 13 taxa in the family Primulaceae chloroplast DNA dataset that was used to provide a secondary calibration for the section** ***Armerina*** **dataset.** (DOCX 81 kb)

## Abbreviations

QTP: Qinghai-Tibet Plateau
ML: Maximum Likelihood
PP: Posterior Probability
PCA: Principal Component Analysis
EPIC: Exon-primed-intron-crossing
PCR: Polymerase chain reactions
AIC: Akaike information criterion
S-DIVA: Statistical Dispersal Vicariance Analysis
MCC: Maximum Clade Credibility
