# Supplementary material for: Phylogeny and biogeography of Primula sect. Armerina: implications for plant evolution under climate change and the uplift of the Qinghai-Tibet Plateau
Source: BMC Evol Biol. 2015 Aug 16;15:161. doi: 10.1186/s12862-015-0445-7 (PMC4537560; doi:10.1186/s12862-015-0445-7)
Supplement: Additional file 1: — The 19 bioclimatic variables for each samples of the three groups used for the niche models. (DOCX 153 kb) [file 12862_2015_445_MOESM1_ESM.docx]

**Additional file 1**

List of samples used in the present study.

| Taxon ID | Location | Longitude (°) | Latitude (°) | Altitude (m) |
| --- | --- | --- | --- | --- |
| *Primula nutans1* | Ruoergai SC | 102.95 | 33.5853 | 3442 |
| *Primula nutans2* | Qilian QH | 100.3797 | 38.074 | 2972 |
| *Primula nutans3* | Zhuoni GS | 102.982 | 34.4057 | 3240 |
| *Primula nutans4* | Hualong QH | 102.2295 | 36.215 | 3523 |
| *Primula nutans5* | Finnmark, Norway | 23.465 | 69.975 | 11 |
| *Primula nutans6* | Alska, USA | -165.293 | 64.487 | 7 |
| *Primula nutans7* | Oulu, Finland | 24.344 | 54.531 | 0.5 |
| *Primula nutans8* | Muren, Mongolica | 99.652 | 49.619 | 1352 |
| *Primula nutans9* | Yukon, Canada | -135.121 | 60.697 | 741 |
| *Primula fasciculata1* | Yushu QH | 96.6558 | 32.874 | 4379 |
| *Primula fasciculata2* | Manigange SC | 98.384 | 32.543 | 4212 |
| *Primula fasciculata3* | Luhuo SC | 100.7143 | 31.719 | 3789 |
| *Primula fasciculata4* | Shujingsi SC | 102.528 | 32.127 | 3478 |
| *Primula fasciculata5* | Ranwuzhen T | 96.7421 | 29.58 | 4349 |
| *Primula fasciculata6* | Bangda T | 97.2879 | 30.207 | 4165 |
| *Primula fasciculata7* | Riwoqe T | 96.8453 | 31.1143 | 3888 |
| *Primula fasciculata8* | Nangqian QH | 96.4846 | 32.0185 | 3825 |
| *Primula fasciculata9* | Gonghe QH | 99.301 | 35.386 | 4444 |
| *Primula fasciculata10* | Ruduoxiang T | 92.348 | 29.803 | 4845 |
| *Primula fasciculata11* | Anduo T | 91.707 | 32.001 | 4684 |
| *Primula fasciculata12* | Budongquan QH | 94.131 | 35.068 | 4476 |
| *Primula fasciculata13* | Gonghe QH | 98.703 | 35.066 | 4476 |
| *Primula fasciculata14* | Ningzhongxiang T | 91.407 | 30.559 | 4465 |
| *Primula fasciculata15* | Dangxiong T | 91.69 | 31.055 | 4686 |
| *Primula fasciculata16* | Sangxiong T | 91.806 | 31.27 | 4592 |
| *Primula fasciculata17* | Naqu T | 91.739 | 31.629 | 4548 |
| *Primula tibetica1* | Gar T | 80.6783 | 31.2655 | 4561 |
| *Primula tibetica2* | Nyalam T | 86.1211 | 28.4037 | 4558 |
| *Primula tibetica3* | Kangmar T | 89.65 | 28.63 | 4400 |
| *Primula tibetica4* | Zhongdazhen T | 92.8119 | 29.0605 | 3125 |
| *Primula tibetica5* | Lulangzhen T | 94.7376 | 29.7631 | 3373 |
| *Primula tibetica6* | Qushui T | 90.2643 | 29.3388 | 3748 |
| *Primula tibetica7* | Huoerxiang T | 81.6809 | 30.7329 | 4641 |
| *Primula tibetica8* | Angren T | 87.33 | 29.212 | 4315 |
| *Primula involucrata1* | Maerkang SC | 100.749 | 31.783 | 3945 m |
| *Primula involucrata2* | Hongyuan SC | 102.528 | 32.127 | 3464 m |
| *Primula involucrata3* | Songpan SC | 103.453 | 32.907 | 3425 |
| *Primula involucrata4* | Songpan SC | 103.453 | 32.907 | 3425 |
| *Primula pamirica1* | NA | NA | NA | NA |
| *Primula pamirica2* | NA | NA | NA | NA |
| *Primula pamirica3* | NA | NA | NA | NA |
| *Primula conspersa1* | Kangle GS | 103.748 | 34.937 | 2806 |
| *Primula conspersa2* | Hualong QH | 102.229 | 36.215 | 3780 |
| *Primula conspersa3* | Ledu QH | 102.277 | 36.667 | 4025 |
| *Primula gemmifera1* | Hongyuan SC | 102.628 | 32.91 | 3510 |
| *Primula gemmifera2* | Luhuo SC | 100.714 | 31.719 | 3228 m |
| *Primula gemmifera3* | Diebu GS | 102.982 | 34.406 | 3145 m |
| *Primula gemmifera4* | Maqin QH | 100.705 | 34.594 | 3345 |
| *Primula gemmifera5* | Maqin QH | 100.63 | 34.65 | 3647 |
| *Primula zambalensis1* | NA | NA | NA | NA |
| *Primula zambalensis2* | NA | NA | NA | NA |
| *Primula pumilio1* | Dege SC | 98.487 | 31.622 | 4288 |
| *Primula pumilio2* | Changdu T | 97.205 | 30.674 | 4482 |
| *Primula pumilio3* | Nyalam T | 86.341 | 28.782 | 4418 |
| *Primula pumilio4* | Lazi T | 85.499 | 29.383 | 4646 |
| *Primula pumilio5* | Riduoxiang T | 92.067 | 29.706 | 4166 |
| *Primula reticulata* | Nyalam T | 85.983 | 28.152 | 3852 |
| *Primula alpicola* | Gyaca T | 92.682 | 29.101 | 3185 |
| *Primula sikkimensis* | Baxoi T | 97.598 | 30.115 | 3871 |
| *Primula egaliksensis* | NA | NA | NA | NA |
| *Primula mistassinica* | NA | NA | NA | NA |
| *Primula pinnatifida* | NA | NA | NA | NA |
| *Primula farinosa* | NA | NA | NA | NA |
