# Supplementary material for: Phylogeny and biogeography of Primula sect. Armerina: implications for plant evolution under climate change and the uplift of the Qinghai-Tibet Plateau
Source: BMC Evol Biol. 2015 Aug 16;15:161. doi: 10.1186/s12862-015-0445-7 (PMC4537560; doi:10.1186/s12862-015-0445-7)
Supplement: Additional file 2: — The PC1 and PC2 values summarized from the 19 bioclimatic variables used for the niche models. (DOCX 79 kb) [file 12862_2015_445_MOESM2_ESM.docx]

**Additional file 2**

GenBank accession numbers for DNA sequence data of the 13 taxa in the family Primulaceae chloroplast DNA dataset that was used to provide a secondary calibration for the section *Armerina* dataset.

| Taxon | *rbc*L | *mat*K | *ndh*F | *rps*16 | *rpl*16 | *trn*L-F |
| --- | --- | --- | --- | --- | --- | --- |
| *Androsace septentrionalis* | AF394963 | JN966080 | AF421116 | FJ786608 | AF402555 | AY274959 |
| *Soldanella montana* | U96943 | AY647533 | AF213761 | AM990487 | DQ378517 | DQ378606; DQ378832 |
| *Cortusa matthioli* | Z83146 | AY647522 | AF213739 | AY647597 | AY528555 | AY622987 |
| *Dodecatheon meadia* | U96658 | AY647482 | AF213741 | AY647557 | AY528528 | AY647628; AY647698 |
| *Primula fasciculata* | JF943645 | DQ378329 | NA | DQ379953 | EF218336 | DQ378734; DQ378544 |
| *Primula edelbergii* | AF213797 | AY647528 | AF213754 | AY647603 | AF402452 | AY647673; AY647743 |
| *Primula cortusoides* | AF213800 | DQ378412 | AF213753 | NA | DQ378506 | DQ378817; DQ378594 |
| *Primula alpicola* | AF394987 | FJ828606 | NA | FJ786574 | This study | FJ794205 |
| *Primula cuneifolia* | AF394962 | AY647502 | NA | AY647577 | AF402534 | AY647647; AY647717 |
| *Primula gaubaeana* | AF213798 | DQ378297 | AF213755 | NA | DQ378433 | DQ378702; DQ378522 |
| *Primula involucrata* | AF394974 | DQ378328 | NA | DQ379954 | DQ378454 | DQ379777 |
| *Primula palinuri* | AF213801 | AY647489 | AF213756 | AY647564 | AF402532 | AY647634; AY647704 |
| *Primula sikkimensis* | AF213799 | FJ828642 | AF213758 | FJ786599 | This study | FJ794231 |
