# Supplementary material for: Phylogeny and biogeography of Primula sect. Armerina: implications for plant evolution under climate change and the uplift of the Qinghai-Tibet Plateau
Source: BMC Evol Biol. 2015 Aug 16;15:161. doi: 10.1186/s12862-015-0445-7 (PMC4537560; doi:10.1186/s12862-015-0445-7)
Supplement: Additional file 3: — Average AICc weights for each niche evolutionary model. The values are averages across estimations done on 100 trees with 10 stochastic maps. Quantiles (reported in brackets below each average AICc weight) are calculated as 2.5 % and 97.5 % from the distribution of AICc weights based on 100 trees, with 10 stochastic maps. The bold values show the best-fit models for each set and each PC axis. (DOCX 86 kb) [file 12862_2015_445_MOESM3_ESM.docx]

**Additional file 3**

The 19 bioclimatic variables for each samples of the three groups used for the niche models.

| Taxon | bio1 | bio2 | bio3 | bio4 | bio5 | bio6 | bio7 | bio8 | bio9 | bio10 | bio11 | bio12 | bio13 | bio14 | bio15 | bio16 | bio17 | bio18 | bio19 |
| --- | --- | --- | --- | --- | --- | --- | --- | --- | --- | --- | --- | --- | --- | --- | --- | --- | --- | --- | --- |
| *P. nutans1* | 18 | 151 | 40 | 6999 | 174 | -196 | 370 | 96 | -80 | 100 | -80 | 660 | 122 | 3 | 82 | 347 | 14 | 333 | 14 |
| *P. nutans2* | 9 | 132 | 34 | 8822 | 186 | -201 | 387 | 116 | -111 | 116 | -111 | 333 | 77 | 1 | 101 | 211 | 4 | 211 | 4 |
| *P. nutans3* | 22 | 145 | 39 | 7159 | 179 | -187 | 366 | 103 | -78 | 107 | -78 | 617 | 118 | 2 | 84 | 328 | 12 | 319 | 12 |
| *P .nutans4* | -6 | 127 | 36 | 7552 | 150 | -201 | 351 | 86 | -109 | 86 | -109 | 526 | 112 | 2 | 92 | 299 | 9 | 299 | 9 |
| *P. fasciculata1* | -4 | 145 | 41 | 7115 | 154 | -194 | 348 | 82 | -91 | 82 | -99 | 494 | 110 | 2 | 97 | 302 | 10 | 302 | 11 |
| *P. fasciculata2* | 8 | 148 | 42 | 6930 | 162 | -183 | 345 | 91 | -77 | 91 | -85 | 534 | 117 | 3 | 97 | 320 | 10 | 320 | 11 |
| *P. fasciculata3* | 35 | 156 | 44 | 6635 | 184 | -164 | 348 | 114 | -47 | 114 | -56 | 679 | 140 | 4 | 93 | 387 | 14 | 387 | 15 |
| *P. fasciculata4* | 45 | 154 | 45 | 6137 | 193 | -147 | 340 | 119 | -39 | 119 | -39 | 803 | 148 | 4 | 83 | 407 | 18 | 407 | 18 |
| *P. fasciculata5* | 23 | 128 | 44 | 5693 | 152 | -136 | 288 | 92 | -42 | 92 | -52 | 601 | 132 | 3 | 89 | 353 | 15 | 353 | 16 |
| *P. fasciculata6* | 32 | 138 | 44 | 6015 | 171 | -137 | 308 | 106 | -38 | 106 | -47 | 538 | 120 | 2 | 95 | 324 | 10 | 324 | 11 |
| *P. fasciculata7* | 46 | 149 | 44 | 6488 | 197 | -137 | 334 | 126 | -32 | 126 | -40 | 514 | 114 | 2 | 97 | 313 | 10 | 313 | 10 |
| *P. fasciculata8* | 39 | 147 | 42 | 6827 | 196 | -147 | 343 | 122 | -45 | 122 | -53 | 537 | 118 | 3 | 98 | 329 | 11 | 329 | 11 |
| *P. fasciculata9* | -49 | 136 | 36 | 8199 | 116 | -256 | 372 | 49 | -150 | 49 | -159 | 388 | 86 | 2 | 93 | 232 | 9 | 232 | 9 |
| *P. fasciculata10* | -2 | 150 | 44 | 6763 | 145 | -195 | 340 | 82 | -90 | 82 | -90 | 289 | 81 | 0 | 118 | 206 | 2 | 206 | 2 |
| *P. fasciculata11* | -24 | 150 | 40 | 7918 | 143 | -229 | 372 | 74 | -117 | 74 | -127 | 372 | 98 | 2 | 110 | 255 | 6 | 255 | 6 |
| *P. fasciculata12* | -40 | 141 | 37 | 8298 | 135 | -239 | 374 | 64 | -138 | 64 | -148 | 252 | 65 | 1 | 103 | 166 | 3 | 166 | 4 |
| *P. fasciculata13* | -46 | 135 | 36 | 8234 | 121 | -249 | 370 | 54 | -146 | 54 | -156 | 364 | 80 | 3 | 91 | 217 | 11 | 217 | 12 |
| *P. fasciculata14* | 12 | 148 | 42 | 7168 | 166 | -183 | 349 | 101 | -66 | 101 | -82 | 338 | 95 | 0 | 122 | 248 | 2 | 248 | 2 |
| *P. fasciculata15* | -11 | 150 | 41 | 7538 | 149 | -214 | 363 | 82 | -93 | 82 | -110 | 357 | 96 | 1 | 117 | 251 | 4 | 251 | 4 |
| *P. fasciculata16* | -10 | 152 | 41 | 7663 | 153 | -216 | 369 | 84 | -93 | 84 | -111 | 382 | 100 | 1 | 115 | 264 | 5 | 264 | 5 |
| *P. fasciculata17* | -12 | 152 | 40 | 7850 | 152 | -220 | 372 | 84 | -106 | 84 | -116 | 388 | 101 | 2 | 112 | 266 | 6 | 266 | 6 |
| *P. tibetica1* | -2 | 127 | 40 | 6932 | 153 | -162 | 315 | 82 | -48 | 88 | -90 | 513 | 117 | 8 | 78 | 280 | 47 | 256 | 89 |
| *P. tibetica2* | 7 | 140 | 47 | 5639 | 140 | -157 | 297 | 72 | -56 | 77 | -67 | 373 | 112 | 1 | 116 | 263 | 13 | 255 | 20 |
| *P. tibetica3* | 25 | 147 | 44 | 6446 | 166 | -165 | 331 | 104 | -60 | 104 | -60 | 273 | 84 | 0 | 131 | 205 | 0 | 205 | 0 |
| *P. tibetica4* | 97 | 142 | 45 | 5937 | 230 | -79 | 309 | 169 | 17 | 169 | 17 | 564 | 136 | 1 | 103 | 366 | 4 | 366 | 4 |
| *P. tibetica5* | 77 | 127 | 44 | 5620 | 205 | -80 | 285 | 145 | 1 | 145 | 1 | 629 | 128 | 2 | 91 | 361 | 9 | 361 | 9 |
| *P. tibetica6* | 68 | 147 | 44 | 6444 | 215 | -117 | 332 | 146 | -18 | 146 | -18 | 401 | 118 | 0 | 127 | 301 | 2 | 301 | 2 |
| *P. tibetica7* | -1 | 118 | 42 | 6049 | 136 | -142 | 278 | 71 | -42 | 76 | -78 | 607 | 132 | 6 | 73 | 318 | 53 | 292 | 104 |
| *P. tibetica8* | 28 | 161 | 44 | 6941 | 190 | -168 | 358 | 109 | -51 | 116 | -61 | 295 | 108 | 1 | 142 | 234 | 5 | 231 | 6 |
