# Supplementary material for: Phylogeny and biogeography of Primula sect. Armerina: implications for plant evolution under climate change and the uplift of the Qinghai-Tibet Plateau
Source: BMC Evol Biol. 2015 Aug 16;15:161. doi: 10.1186/s12862-015-0445-7 (PMC4537560; doi:10.1186/s12862-015-0445-7)
Supplement: Additional file 4: — Model fit and estimates parameters of supported OUwie models for the four group-sets. Parameter estimates are averages across estimations done on 100 trees with 10 stochastic maps. Quantiles (reported in brackets) are calculated as 2.5 % and 97.5 % from the distribution of AICc weights based on 100 trees, with 10 stochastic maps. F1, F2 and NT are three groups defined based on the chloroplast tree (see Fig. 4). (DOCX 119 kb) [file 12862_2015_445_MOESM4_ESM.docx]

**Additional file 4**

The PC1 and PC2 values summarized from the 19 bioclimatic variables used for the niche models.

| Taxon | PC1 | PC2 |
| --- | --- | --- |
| *P. nutans1* | -0.072987263 | 0.818225907 |
| *P. nutans2* | -3.623615128 | -1.73813949 |
| *P. nutans3* | -0.169264836 | 0.197712339 |
| *P .nutans4* | -2.319272587 | 1.129968328 |
| *P. fasciculata1* | -1.699157334 | 0.505960085 |
| *P. fasciculata2* | -0.551564792 | 0.391594428 |
| *P. fasciculata3* | 2.299355921 | 0.288055407 |
| *P. fasciculata4* | 3.705752876 | 0.726808613 |
| *P. fasciculata5* | 1.975568723 | 1.504731945 |
| *P. fasciculata6* | 1.693138838 | -0.273509691 |
| *P. fasciculata7* | 2.0124741 | -1.590323883 |
| *P. fasciculata8* | 1.573041748 | -0.925288342 |
| *P. fasciculata9* | -6.290940062 | 1.896501278 |
| *P. fasciculata10* | -3.097823056 | -1.857471263 |
| *P. fasciculata11* | -4.124824061 | -0.051416453 |
| *P. fasciculata12* | -6.587520087 | -0.272302872 |
| *P. fasciculata13* | -6.125345233 | 1.977062439 |
| *P. fasciculata14* | -1.815672042 | -2.336904789 |
| *P. fasciculata15* | -3.319090757 | -1.011419344 |
| *P. fasciculata16* | -3.128571506 | -0.941481975 |
| *P. fasciculata17* | -3.338417694 | -0.489516616 |
| *P. tibetica1* | 0.272296503 | 5.387649118 |
| *P. tibetica2* | -0.475406553 | -0.022181919 |
| *P. tibetica3* | -1.44652084 | -3.201364962 |
| *P. tibetica4* | 5.898031308 | -3.556249518 |
| *P. tibetica5* | 5.126030174 | -1.380167719 |
| *P. tibetica6* | 2.638877346 | -4.131154209 |
| *P. tibetica7* | 1.472947157 | 6.579217571 |
| *P. tibetica8* | -0.740077436 | -3.437614472 |
