# Supplementary material for: Phylogeny and biogeography of Primula sect. Armerina: implications for plant evolution under climate change and the uplift of the Qinghai-Tibet Plateau
Source: BMC Evol Biol. 2015 Aug 16;15:161. doi: 10.1186/s12862-015-0445-7 (PMC4537560; doi:10.1186/s12862-015-0445-7)
Supplement: Additional file 6: — GenBank accession numbers for DNA sequence data of the 13 taxa in the family Primulaceae chloroplast DNA dataset that was used to provide a secondary calibration for the section Armerina dataset. (DOCX 81 kb) [file 12862_2015_445_MOESM6_ESM.docx]

**Additional file 6**

Model fit and estimated parameters of supported OUwie models for the four group-sets.

| Model | | AICc weight |  | θ | | |  | σ^2^ | | |  | α | | | | |
| --- | --- | --- | --- | --- | --- | --- | --- | --- | --- | --- | --- | --- | --- | --- | --- | --- |
| *SET1 (F1/F2 vs. NT)* | | |  | ***F1/F2*** | ***NT*** |  |  | ***F1/F2*** | ***NT*** |  |  | ***F1/F2 NT*** | | | | |
| PC1 | OU1 | 0.461  (0.454-0.468) |  | -0.09 (-0.1, -0.06) | | |  | 106 (97.7, 109) | | |  | 5.24 (4.95, 5.45) | | | | |
|  | OUM | 0.251  (0.247-0.256) |  | -1.06  (-1.08, -1.04) | 0.95  (0.93, 0.98) |  |  | 120.7 (114, 125.5) | | |  | 6.7 (6.28, 6.93) | | | | |
| PC2 | OUMV | 0.972  (0.970-0.973) |  | -0.15  (-0.15, -0.14) | -0.32  (-0.33, -0.31) |  |  | 112.7  (98.26, 136.2) | 912  (765, 996) |  |  | 39.77 (33.62, 46.12) | | | | |
| *SET2 (F1 vs. F2 vs. NT)* | | |  | ***F1*** | ***F2*** | ***NT*** |  | ***F1*** | ***F2*** | ***NT*** |  | ***F1 F2 NT*** | | | | |
| PC1 | OU1 | 0.355  (0.344-0.366) |  | -0.12 (-0.14, -0.11) | | |  | 118 (115, 127) | | |  | 5.88 (5.57, 6.07) | | | | |
|  | OUMV | 0.285  (0.277-0.293) |  | -0.36  (-0.39, -0.32) | -3.14  (-3.14, -3.12) | 0.92  (0.91, 0.95) |  | 219  (213, 229) | 14.02  (13.56, 14.75) | 171  (163, 176) |  | 10.41 (10.16, 10.67) | | | | |
|  | OUMA | 0.183  (0.175-0.191) |  | -0.3  (-0.35, -0.26) | -3.25  (-3.3, -3.22) | 0.92  (0.9, 0.94) |  | 157 (148.5, 166) | | |  | 10  (9.8, 11) | 7  (6.6, 7.4) | | | 11  (10, 11) |
|  | OUM | 0.116  (0.113-0.118) |  | -0.25  (-0.3, -0.2) | -3.32  (-3.37, -3.28) | 0.93  (0.91, 0.95) |  | 166 (156, 188) |  |  |  | 10 (9.5, 10.4) | | | | |
| PC2 | OUMV | 0.933  (0.930-0.977) |  | 0.2  (0.2, 0.2) | -0.97  (-0.97, -0.97) | -0.31  (-0.32, -0.29) |  | 166  (129, 223) | 69.1  (52.5, 105) | 1350  (1023, 1671) |  | 58.8 (43.8, 77.3) | | | | |
| *SET3 (F1 vs. F2/NT)* | | |  | ***F1*** | ***F2/NT*** |  |  | ***F1*** | ***F2/NT*** |  |  | ***F1 F2/NT*** | | | | |
| PC1 | OU1 | 0.658  (0.654-0.663) |  | -0.14 (-0.16, -0.13) | | |  | 118 (115, 123) | | |  | 5.84 (5.44, 6.17) | | | | |
| PC2 | OUMV | 0.784  (0.779-0.789) |  | 0.2  (0.2, 0.2) | -0.48  (-0.48, -0.48) |  |  | 432  (358, 584) | 2388  (1978, 3321) |  |  | 150 (124, 202) | | | | |
| *SET4 (F2 vs. F1/NT)* | | |  | ***F2*** | ***F1/NT*** |  |  | ***F2*** | ***F1/NT*** |  |  | ***F2 F1/NT*** | | | | |
| PC1 | OUMV | 0.478  (0.471-0.486) |  | -3.15  (-3.15, -3.14) | 0.38  (0.36, 0.41) |  |  | 10.42  (9.91, 10.64) | 146  (139, 153) |  |  | 7.17 (6.83, 7.63) | | | | |
|  | OUMA | 0.269  (0.260-0.278) |  | -3.4  (-3.47, -3.32) | 0.3  (0.28, 0.33) |  |  | 144.6 (136.2, 154.5) | | |  | 5.65  (5.45, 6.08) | |  | 8.38  (8.01, 8.87) | |
| PC2 | OUMV | 0.636  0.631-0.640) |  | -0.97  (-0.97, -0.97) | -0.05  (-0,05, -0,04) |  |  | 73.84  (60.61, 102) | 781  (621, 1059) |  |  | 62.79 (50.84, 86.26) | | | | |
|  | OU1 | 0.268  (0.265-0.271) |  | -0.21 (-0.21, -0.21) | | |  | 566 (497, 696) | | |  | 52.82 (44.72, 65.1) | | | | |

Parameter estimates are averages across estimations done on 100 trees with 10 stochastic maps. Quantiles (reported in brackets below each parameter) are calculated as 2.5% and 97.5% from the distribution of AIC weights based on 100 trees, with 10 stochastic maps. F1, F2 and NT are three groups defined based on the chloroplast tree (see Figure 3).
